# Demystifying the ‘hidden curriculum’ for minoritized graduate students

**DOI:** 10.7554/eLife.94422

**Published:** 2024-03-12

**Authors:** Michael J Hopkins, Brittni N Moore, Jasmin L Jeffery, Andrea S Young

**Affiliations:** 1 Department of Biological Chemistry, Johns Hopkins School of Medicine Baltimore United States; 2 Department of Physiology, Johns Hopkins School of Medicine Baltimore United States; 3 Department of Cell Biology, Johns Hopkins School of Medicine Baltimore United States; 4 Department of Psychiatry and Behavioral Sciences, Johns Hopkins School of Medicine Baltimore United States; 5 Department of Mental Health, Johns Hopkins Bloomberg School of Public Health Baltimore United States

**Keywords:** equity, diversity, inclusion, hidden curriculum, graduate school, education, training, professional development, None

## Abstract

Graduate programs in the biomedical sciences dedicate considerable resources to recruiting students from underrepresented racial and ethnic groups. However, students from these minoritized groups have decreased access to the ‘hidden curriculum’ that must be navigated in order to be successful in graduate school. Here, we describe a student-led initiative at Johns Hopkins University, the Hidden Curriculum Symposium, that is organized to help prepare new students from underrepresented groups for graduate school. Preliminary evidence from surveys suggests that the initiative does increase the preparedness of minoritized students, and we believe this approach could also prove useful at other academic institutions.

## Introduction

The ‘leaky pipeline’ is a metaphor that describes a persistent problem in which certain demographic groups, particularly women and minoritized groups, are more likely than other demographic groups to leave careers in science, technology, engineering and mathematics (Hinton et al., 2020; Hinton and Lambert, 2022; Jackson, 2023; Lin, 2021). In addition to depleting the pool of available talent in these subjects, the leaky pipeline also reduces diversity (Sarraju et al., 2023) and hinders scientific progress (Hofstra et al., 2020). Almost one-third of the students who leave a PhD program do so during their first year (Golde, 1998), so there is a clear opportunity to reduce leaks during the first year of graduate school (Golde, 2000; Golde, 2005).

Academia also has a ‘hidden curriculum’ – a set of unspoken norms and customs related to topics such as selecting a PhD advisor, accessing resources, and resolving interpersonal conflicts (Pensky et al., 2021; Mendoza-Denton et al., 2017). Understanding these norms and customs is crucial for success, but graduate students from underrepresented racial and ethnic groups tend to be less aware of these unwritten rules and must discover them on their own. This lack of knowledge about the hidden curriculum contributes to poor outcomes for these students, including fewer publications (Gibbs et al., 2014), a decreased sense of belonging (Mendoza-Denton et al., 2017), poor mental health (Mendoza-Denton et al., 2017), and being less likely to pursue an academic career (Gibbs et al., 2014; Gibbs et al., 2016). This happens in addition to the structural and systemic racism that these students already face (Hinton et al., 2020; Sarraju et al., 2023; Allen-Ramdial and Campbell, 2014; Mays et al., 2023).

This article reports on efforts to demystify the hidden curriculum for first-year graduate students from underrepresented racial and ethnic groups (URGs) at Johns Hopkins University in the United States. The centerpiece of these efforts is a two-day symposium organized by the Biomedical Scholars Association (BSA) at the university, with support from Johns Hopkins faculty. This symposium has two main objectives: (a) to equip these students with the skills and resources needed to address the challenges they might experience as graduate students from underrepresented groups; and (b) to build and develop support networks among first-year URG students, senior graduate students, postdocs, faculty members, and administrators.

## The Hidden Curriculum Symposium

The first symposium took place in September 2022, near the start of the 2022/2023 academic year, and was designed by current graduate students for incoming first-year students. The first day consisted of discussion-based presentations on four topics: (i) academic success (in both coursework and research); (ii) mental health; (iii) conflict resolution; and (iv) financial literacy. The session formats varied, including PowerPoint presentations, panel discussions, hands-on activities, and case studies. Day one concluded with dinner and a social networking event for the first-year graduate students, which was intended to foster peer-to-peer interactions and to build community among students.

**Figure 1.**
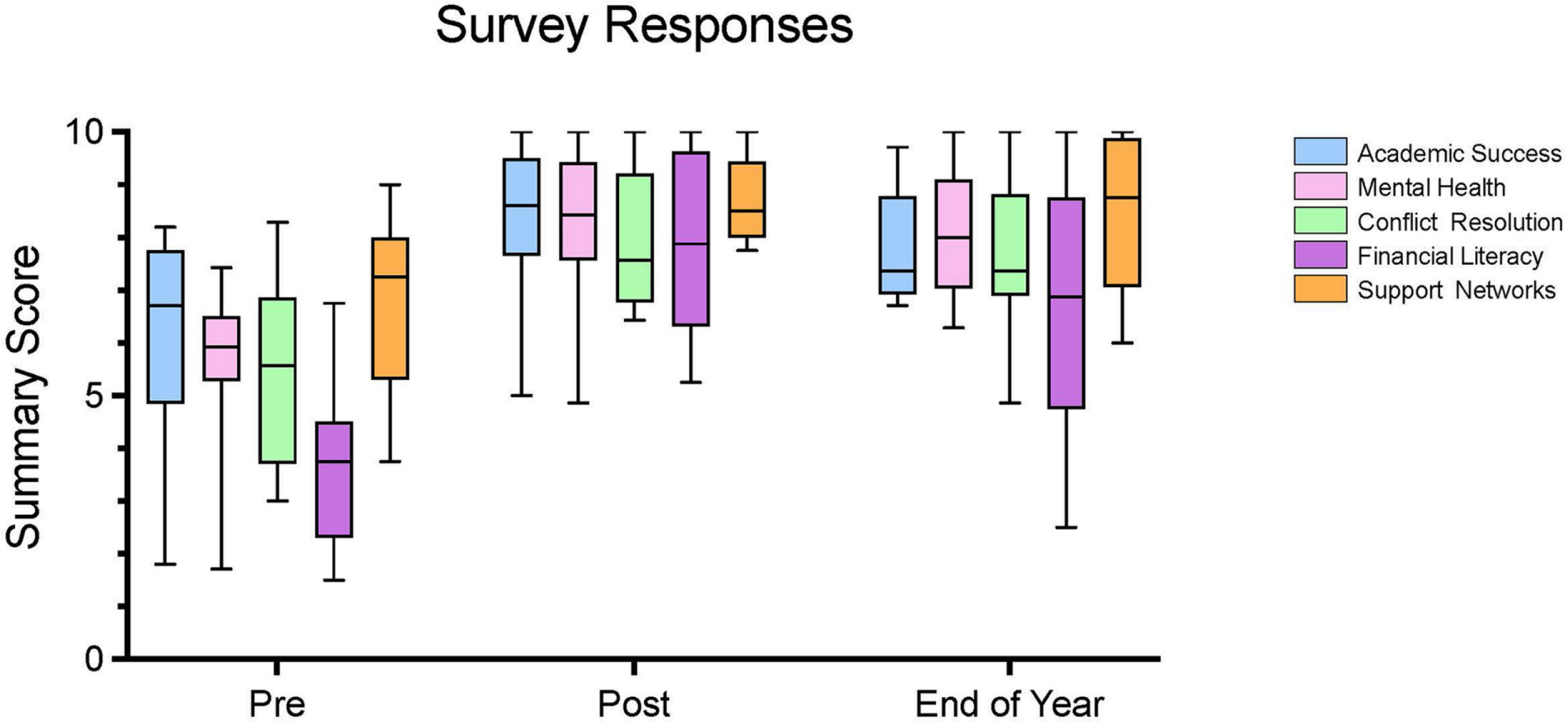
Assessing the impact of the first Hidden Curriculum Symposium over time. The 12 students who attended the first symposium were surveyed seven days before the symposium (Pre; left), immediately after the symposium (Post; middle), and at the end of the first year of graduate school (End of Year; right). The surveys contained questions to determine (on a scale of 1–10; vertical axis) how prepared the students were in five areas: academic success, mental health, conflict resolution, financial literacy, and support networks. See Methods for more information. The summary scores for all five areas were higher after the symposium than they were before, and these increases were statistically significant. The summary scores were lower at the end of the year than they were after the symposium, but only the decrease for financial literacy was statistically significant. Moreover, the summary scores for all five areas at the end of the year were much higher than the scores before the symposium, which suggested the symposium’s impacts were sustained until the end of the year. We also explored if enrolling in graduate school immediately after completing an undergraduate degree (as opposed to, for example, completing a master’s degree) or attending a Minority Serving Institution (MSI) influenced the results, but we did not find any evidence for this (see Table S4 in Supplementary file 1). Each ‘box‘ in these plots indicates the interquartile range (25^th^ to 75^th^ percentile), while the horizontal line running through the box is the 50^th^ percentile (median); the lines extending from each box indicate the full range of the summary scores for that topic.

The second day was a brunch attended by first-year graduate students, other graduate students from URGs, postdoctoral researchers, and faculty (about 50 people in total), followed by an opportunity to network. All first-year students who attended the two days of the symposium were given a personalized ‘graduate school survival kit’ that included practical items (such as flash drives, pens and notebooks) and memorabilia (gift card, backpack, sweatshirt), and took part in a raffle for prizes such as a coffee maker and an iPad. The results of surveys of the 12 students who attended the first symposium suggest that the effects of the symposium were beneficial (Figure 1). Moreover, except for financial literacy (see below), the benefits persisted until the end of the academic year. Informal feedback also suggests that faculty and the wider Johns Hopkins community also benefitted from the symposium.

The second symposium, which took place in September 2023, had a similar format but was attended by 34 first-year students (compared with 12 for the first symposium), and there were about 100 people at the networking event on the second day. Reasons for the increased attendance included better advertising, increased awareness of the symposium via the testimony of those who attended the first event, and the presence of local alumni at the networking event. In addition, the mental health session on day one was led by a licensed Johns Hopkins clinician, and the networking event on day two included a keynote address by an URG faculty member from Johns Hopkins, Dr. Jelani Zarif.

The five areas of the symposium – academic success, mental health; conflict resolution, financial literacy, and support networks – are described in more detail below.

### Academic success

The challenges faced by minoritized graduate students include language barriers, limited interactions with faculty before starting graduate school, and discrimination, and each of these can hinder academic progress (Golde, 2000; Smith et al., 2012; Hamzavi and Brown, 2023). The goal of this session was to ensure URG students began graduate school with the same expectations as every other graduate student, and that they had access to the same tools and resources. This included explicit discussions of procedures and policies new graduate students may be expected to implicitly know (or to figure out), and resources that can help support academic success. For instance, during their initial year, graduate students are expected to achieve a passing grade (B-) in all enrolled courses. The Hidden Curriculum Symposium conveyed this standard to students and gave advice about what to do if expectations were not met, such as guidance on reaching out to the administration to request a course retake.

Other topics covered included: coursework preparation; how to optimize laboratory rotations; and how to select a thesis advisor. Students at Johns Hopkins must complete a minimum of three laboratory rotations, but it is possible to request a fourth rotation during the summer months if the student still needs to find a lab and/or thesis advisor that is a good fit for them. It is crucial for all students – and especially so for minoritized students – to carefully choose their advisor and lab, and part of this decision-making process involves having the flexibility to explore all available options.

In addition, we provided tools for building study groups, discussed how to balance classes and lab work, and provided students with details of various university resources (such as tutoring services that offer peer study groups, additional notes and materials, and practice exams; disability accommodations; and advice on how to report abuse). Finally, the session ended with a panel discussion during which graduate students provided a wide range of perspectives and opinions.

### Mental health

Graduate school can take a toll on the mental health of research students, and it has been reported that mental health disorders like anxiety and depression are six times higher among biomedical PhD students than the general population (Evans et al., 2018). Moreover, poor mental health can compound the problems caused by various forms of discrimination, such as systemic racism, sexism, homophobia, ableism, and discrimination based on social class (Schad et al., 2022).

Our second session aimed to demystify the ‘hidden curriculum’ of mental health by helping students to recognize stressors, to identify signs of problems with mental health, and to access university resources. For example, stressors can manifest as excessive alcohol or substance use, withdrawal from interpersonal relationships, or engagement in risky behaviors. This session reviewed numerous mental health resources that Johns Hopkins provides students, including a 24/7 crisis hotline, access to counseling from in-network mental health professionals, and referrals to out-of-network providers.

Modeled from the Johns Hopkins Student Well-Being Center, this session included a discussion of the ‘seven pillars of wellness’ (Stoewen, 2017) – physical wellness, emotional wellness, intellectual wellness, social wellness, environmental wellness, occupational wellness, and spiritual wellness. Following this discussion, the students completed an arts and crafts activity during which they created ‘values jars’ that included objects that, for each pillar of wellness, represented a goal that the student hoped to achieve during their first year of graduate school. Students could take the values jars home after the session in order to have a daily reminder to prioritize their wellness.

### Conflict resolution

There is a growing recognition of the importance of conflict resolution during graduate school (Schaller and Gatesman-Ammer, 2022). However, most of the interventions designed to resolve conflict do not consider that an URG student who is attempting to resolve a conflict may also be experiencing discrimination, imposter syndrome or microaggressions. Therefore, when conflict arises, URG students need to know how to properly advocate for their needs while being attentive to how they engage with faculty and staff, taking care to use professional language that is both clear and assertive.

This session focused on acquiring and refining skills related to conflict resolution and utilized anonymous case studies based on real interpersonal conflicts experienced by members of the symposium planning committee during graduate school. Many scenarios were explored, including disagreements among classmates, issues between lab mates, and conflicts with professors. The students were also informed about the relevant university resources.

### Financial literacy

Salaries for biomedical PhD students fall far below the cost of living across the United States (Woolston, 2022), so financial stress – and the impact it has on academic success and mental health – is a fact of life for many graduate students, especially those from underrepresented racial and ethnic groups. This session covered topics such as managing personal finances, securing funding through grants and fellowships, understanding federal and state taxes, and navigating the complexities of earning additional income. Research shows that a lack of financial knowledge can lead to increased stress, a decreased sense of belonging, and limited access to resources – all of which can adversely impact the academic success and well-being of URG graduate students (Eichelberger et al., 2017).

One surprising finding from the surveys of the students who attended the symposium was that they were less confident about financial literacy than they were about the other four areas (Figure 1). One possible explanation for this is that there are more resources to advise students on traditional academic topics (such as which courses to take and how to choose a PhD advisor) than there are on general or non-academic topics. Another possibility is that graduate students from minority groups are disproportionately affected by financial burdens compared to white students (Martin and Dwyer, 2021). Either way, it is clear that URG students entering graduate school would benefit from help with both academic topics and more general topics, such as financial literacy.

### Support networks

Success in graduate school generally requires effective mentorship and networking (Edwards et al., 2022), but URG students tend to have less access to both than their white peers due to implicit bias, a lack of representation, and systemic exclusion (Singleton et al., 2021). The networking activities at the symposium were designed to help first-year students establish relationships with their peers and to explore mentorship opportunities with senior graduate students, postdocs, and professors. We aimed to foster the creation of authentic connections that first-year students could utilize throughout their time in graduate school. Indeed, one student first met their future thesis advisor during the 2022 symposium.

## Future directions

As described above, results from the surveys we have conducted so far suggest that the students who attended the first symposium felt better prepared for graduate school (Figure 1), and we plan to observe the potential impact of the project across various metrics as the initial cohort continues through graduate school (such as the number of successfully completed classes and passing qualifying exams).

Future goals for the Hidden Curriculum project include: increasing participation by first-year graduate students; running more events throughout the year; maintaining the improvement in student preparedness as the students progress through graduate school; updating the content of the symposium to keep it timely and relevant; and continuing to build institutional support through financial investment and faculty participation.

**Figure 2.**
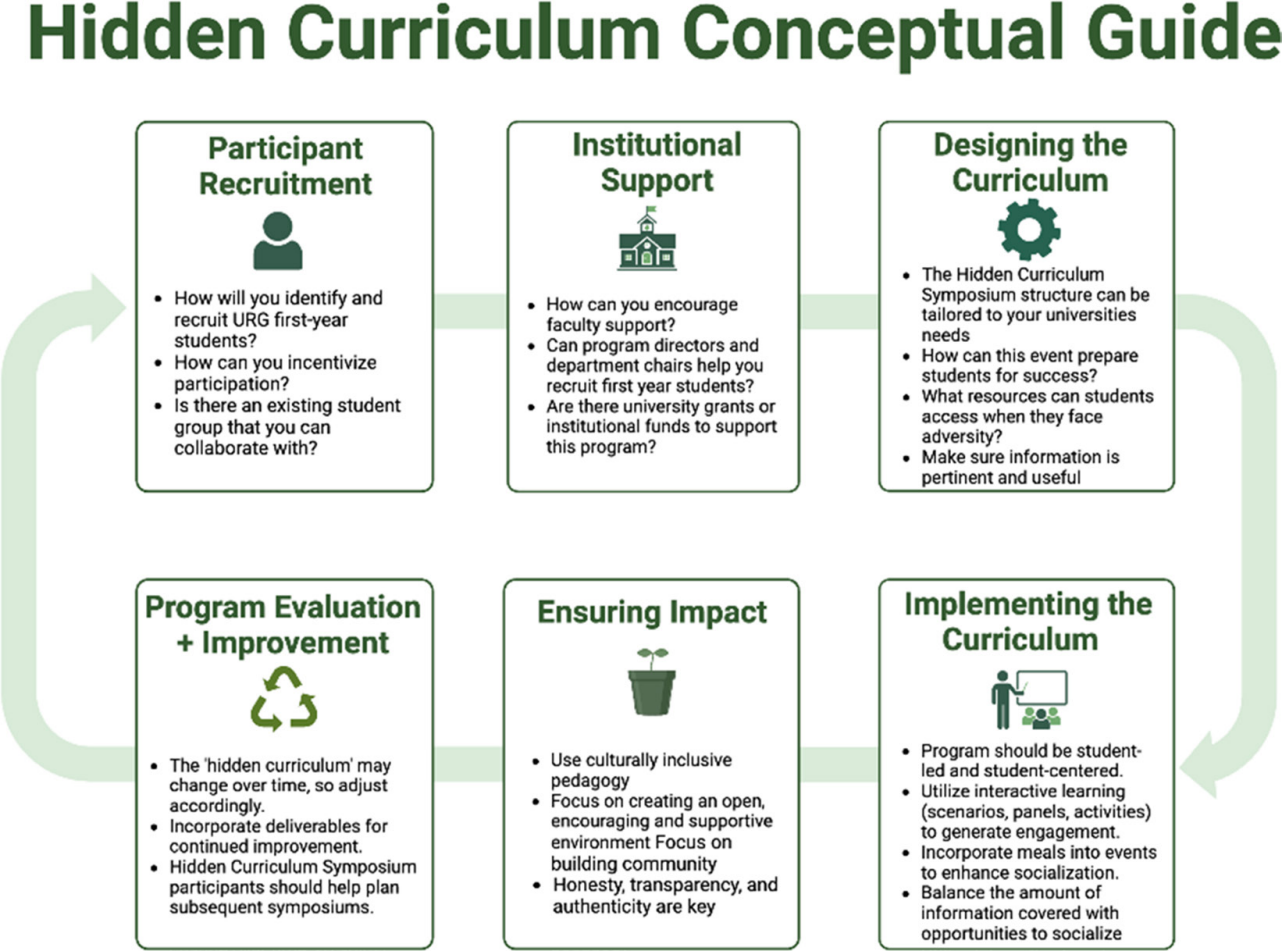
A framework for organizing a symposium on the Hidden Curriculum. The resources created for the 2022 and 2023 Hidden Curriculum symposia at Johns Hopkins are publicly available via Dropbox.

We encourage graduate schools and programs in the life sciences and medicine at other universities to organize their own symposia on the ‘hidden curriculum’ to help address the unique challenges that URG students face in graduate school (Hinton et al., 2020; Sarraju et al., 2023; Allen-Ramdial and Campbell, 2014; Mays et al., 2023), and to reduce the number of students who quit PhDs during their first year (Golde, 1998; Figure 2). To help with this, the resources we created for the first symposium are publicly available via Dropbox. Moreover, it is possible to modify the symposium to make it suitable for new graduate students in other disciplines.

## Methods

In the Fall of 2022, information about the Hidden Curriculum Symposium was sent via institutional email to incoming first-year graduate students as identified by the University Registrar. Of the 59 students contacted, 12 attended the symposium. A majority self-identified as Black/African American (9/12), followed by Latinx/Hispanic (2/12), and Indigenous/Native American (1/12); three-quarters had previously attended a Historically Black College or University (HBCU) or Minority Serving Institution (MSI). Information on the students is given is in Table 1. The study procedures were reviewed and acknowledged by the Johns Hopkins Medicine Institutional Review Board. Caveats to the interpretation of our data include the small sample size (n=12), the absence of a control group of students who did not attend the symposium, and potentially confounding effects (such as rural vs urban, socioeconomic status, and parent’s level of education).

**Table 1.** Characteristics of the students who attended the first Hidden Curriculum Symposium. Note: One student who completed the pre-symposium survey did not attend the symposium and was not included in subsequent analyses.

Variables	Pre-symposium survey	Post-symposium survey	End-of-year survey
Demographics			
Total number of respondents	13	12	12
Age (years; mean ±SD)	23.9±1.33	23.7±1.44	23.7±1.44
			
Race: n (%)			
Black/African American	9 (69.2)	9 (75)	9 (75)
Latinx/Hispanic	2 (15.4)	2 (16.7)	2 (16.7)
Indigenous/Native American	1 (7.7)	1 (8.3)	1 (8.3)
Asian/ Pacific Islander	1 (7.7)	0 (0)	0 (0)
Not Reported	0 (0)	0 (0)	0 (0)
			
Gender: n (%)			
Female	6 (46)	6 (50)	6 (50)
Male	7 (54)	6 (50)	6 (50)
			
Education: n (%)			
Bachelor’s	8 (61.6)	7 (58.4)	7 (58.4)
Master’s	1 (7.6)	1 (8.3)	1 (8.3)
Not Reported	4 (30.8)	4 (33.3)	4 (33.3)
			
Attended HBCU or MSI: n (%)			
Yes	9 (69.2)	9 (75)	9 (75)
No	4 (30.8)	3 (25)	3 (25)

The students who attended the first symposium were surveyed three times. Seven days prior to the symposium, they were sent a pre-survey containing 27 questions (see Table S1 in Supplementary file 1). Immediately after the second day of the symposium, the attendees were sent a post-survey (with the same 27 questions as the pre-survey) to assess the symposium’s effectiveness and to gather data to improve future programming. At the end of their first year, the students were sent an end-of-year survey containing 38 questions (see Table S2 in Supplementary file 1) to provide additional information on the effectiveness of the symposium. For all three surveys, the students were asked to answer each question on a scale ranging from 1 (not effective/not familiar/not confident) to 10 (very effective/very familiar/very confident). All responses were de-identified and anonymized.

The surveys contained between 4 and 7 questions about each of the five areas covered by the symposium (academic success, mental health, conflict resolution, financial literacy, and support networks), so for each area we calculated a summary score which was the mean of the answers for that area. These summary scores demonstrated good to excellent internal consistency as measured by Cronbach’s α (see Table S3 in Supplementary file 1).

Repeated measures ANOVAs examined the differences between the summary scores for the five areas in the three survey responses for the five areas (Figure 1). As an exploratory analysis for this pilot study, we also conducted mixed model ANOVAs examining changes in summary scores while controlling for two variables: (i) whether the students attended a Minority Serving Institution; (ii) whether the students enrolled in graduate school immediately following graduation from an undergraduate institution. No significant effects were observed for these two variables (see Table S4 in Supplementary file 1).

## Data Availability

Given the sensitive nature of the data (that of a small number of students enrolled at a private university), we are unable to make data available.

## Additional files

Supplementary file 1. Tables S1–S4.

MDAR checklist
