# Supplementary material for: Demystifying the ‘hidden curriculum’ for minoritized graduate students
Source: eLife. 2024 Mar 12;13:e94422. doi: 10.7554/eLife.94422 (PMC10932539; doi:10.7554/eLife.94422)
Supplement: Supplementary file 1. [file elife-94422-supp1.docx]

**Table S1. Pre- and post-survey questions.** Survey questions given to study participants before the Hidden Curriculum Symposium and immediately after the Hidden Curriculum Symposium.


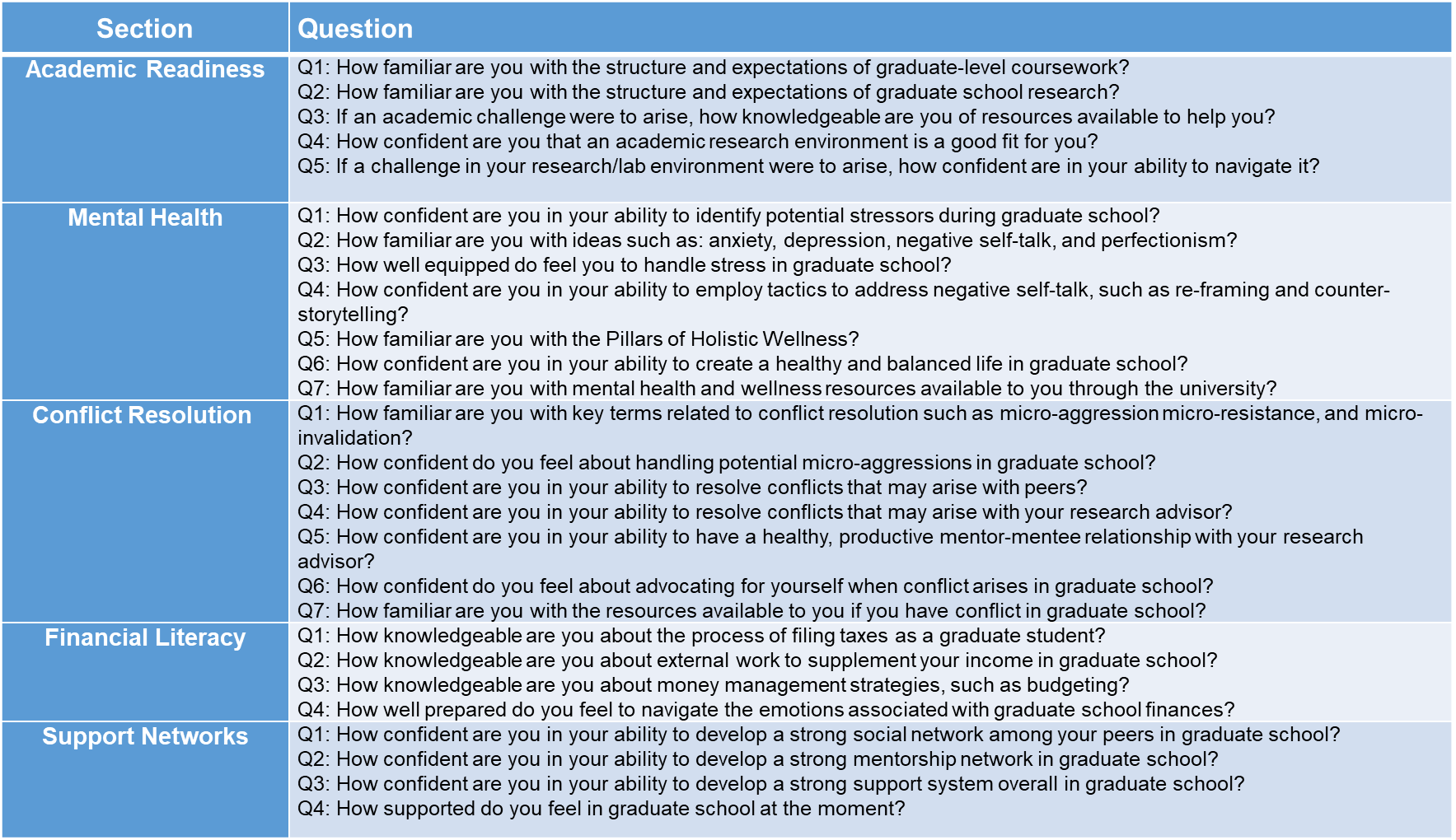


**Table S2. End-of-year survey questions.** Survey questions given to study participants at the end of the first year of graduate school, having completed the Hidden Curriculum Symposium.


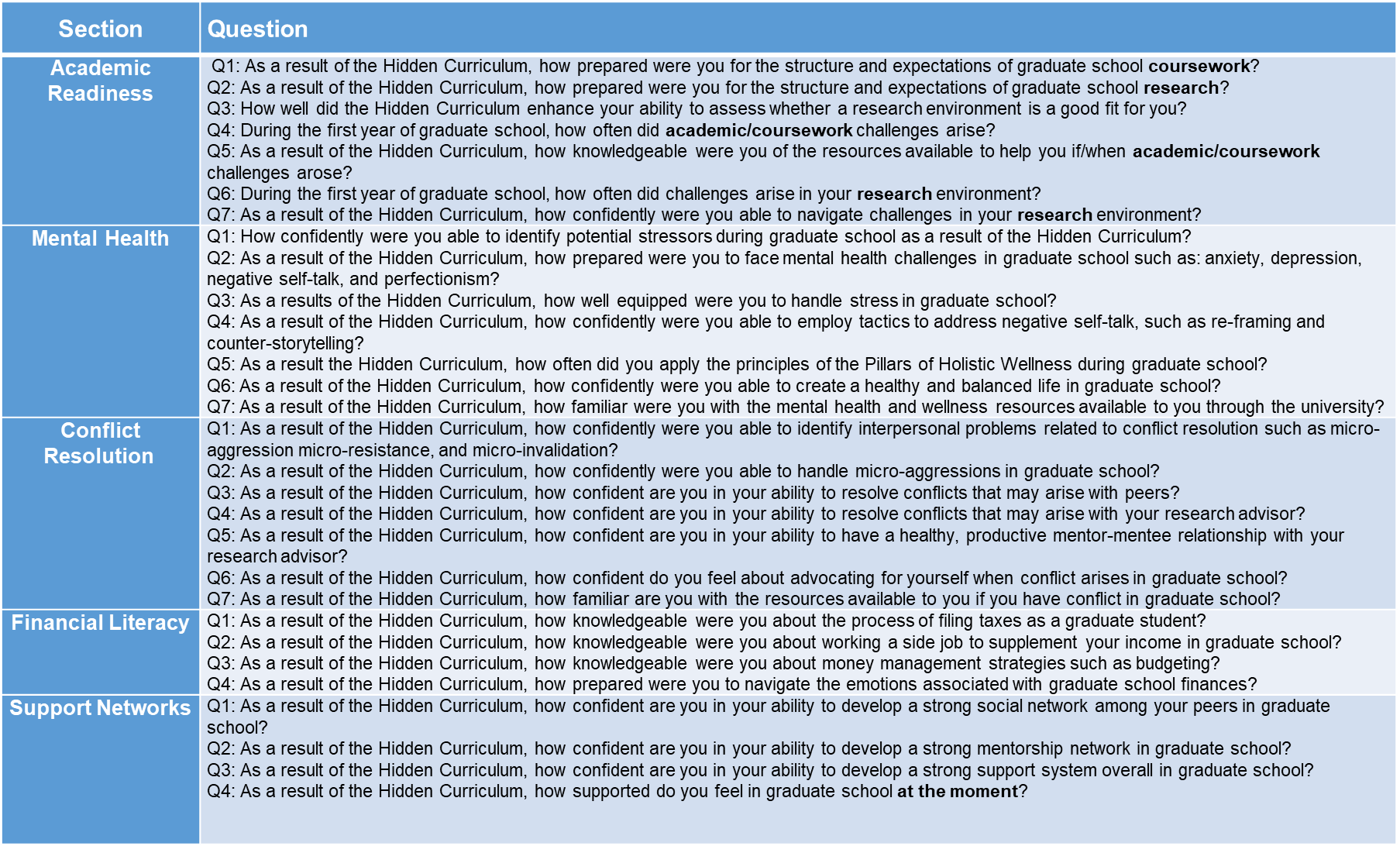


**Table S3. Response to the surveys before the 2022 symposium (pre), immediately after the 2022 symposium (post), and at the end of the 2022/2023 academic year.** For each of the five areas covered by the surveys (column 1), this table contains the number of questions (column 2), the value of Cronbach α (which is a measure of internal consistency), the mean value of the summary score (column 4), and the standard deviation (column 5). Note that Figure 1 displays median values rather than the mean values shown here.

| Area | # of questions | Cronbach’s α | *M* | *SD* |
| --- | --- | --- | --- | --- |
| Academic Success |  |  |  |  |
| Pre | 5 | .883 | 6.2 | 1.8 |
| Post | 5 | .938 | 8.3 | 1.6 |
| End of year | 7 | .790 | 7.8 | 1.0 |
| Mental Health |  |  |  |  |
| Pre | 7 | .816 | 5.6 | 1.5 |
| Post | 7 | .914 | 8.2 | 1.5 |
| End of year | 7 | .824 | 8.0 | 1.2 |
| Conflict Resolution |  |  |  |  |
| Pre | 7 | .858 | 5.5 | 1.7 |
| Post | 7 | .955 | 7.9 | 1.3 |
| End of year | 7 | .955 | 7.6 | 1.5 |
| Financial Literacy |  |  |  |  |
| Pre | 4 | .715 | 3.8 | 1.6 |
| Post | 4 | .896 | 7.9 | 1.7 |
| End of year | 4 | .968 | 6.7 | 2.4 |
| Support Network |  |  |  |  |
| Pre | 4 | .764 | 6.8 | 1.6 |
| Post | 4 | .671 | 8.7 | 0.8 |
| End of year | 4 | .890 | 8.5 | 1.4 |

**Table S4. Results of repeated measure ANOVAs examining the Hidden Curriculum Symposium’s associations with student outcomes over time in unadjusted and adjusted models.**

|  | Unadjusted Model | | | | Adjusted Model | | | |
| --- | --- | --- | --- | --- | --- | --- | --- | --- |
| Outcome | *F_time_*  *df*: 2,22 | *p value* (time) | *M_diff_ (SE)* | *p value* (*M_diff_*) | *F_time_*  *df*: 2,18 | *p value* (time) | *M_diff_ (SE)* | *p value* (*M_diff_*) |
| Academic Success | **6.2** | **.007** |  |  | 3.1 | .069 |  |  |
| Pre-post |  |  | **-2.1 (0.6)** | **.007** |  |  | **-1.8 (0.8)** | **.045** |
| Pre-end of year |  |  | **-1.6 (0.7)** | **.035** |  |  | -0.9 (0.7) | .239 |
| Post-end of year |  |  | 0.5 (0.6) | .426 |  |  | 0.9 (0.7) | .211 |
| Mental Health | **11.5** | **<.001** |  |  | **6.5** | **.008** |  |  |
| Pre-post |  |  | **-2.6 (0.6)** | **.001** |  |  | **-2.4 (0.8)** | **.011** |
| Pre-end of year |  |  | **-2.4 (0.7)** | **.004** |  |  | **-2.2 (0.8)** | **.027** |
| Post-end of year |  |  | 0.2 (0.5) | .690 |  |  | 0.3 (0.6) | .637 |
| Conflict Resolution | **10.1** | **<.001** |  |  | **5.0** | **.019** |  |  |
| Pre-post |  |  | **-2.5 (0.6)** | **.002** |  |  | **-2.2 (0.8)** | **.020** |
| Pre-end of year |  |  | **-2.2 (0.8)** | **.015** |  |  | -1.6 (0.9) | .100 |
| Post-end of year |  |  | 0.3 (0.4) | .422 |  |  | 0.6 (0.4) | .195 |
| Financial Literacy | **21.2** | **<.001** |  |  | **11.8** | **<.001** |  |  |
| Pre-post |  |  | **-4.0 (0.6)** | **<.001** |  |  | **-3.8 (0.8)** | **<.001** |
| Pre-end of year |  |  | **-2.9 (0.8)** | **.005** |  |  | **-2.5 (1.0)** | **.035** |
| Post-end of year |  |  | **1.2 (0.4)** | **.013** |  |  | **1.3 (0.5)** | **.028** |
| Support Network | **7.6** | **.003** |  |  | **3.7** | **.04** |  |  |
| Pre-post |  |  | **-1.9 (0.5)** | **.004** |  |  | **-1.7 (0.7)** | **.029** |
| Pre-end of year |  |  | **-1.7 (0.6)** | **.023** |  |  | -1.2 (0.8) | .142 |
| Post-end of year |  |  | 0.2 (0.4) | .622 |  |  | 0.5 (0.5) | .342 |

**Note**: The unadjusted models examined only the within subjects effect of participating in the Hidden Curriculum event over time (pre-Hidden Curriculum, post-Hidden Curriculum, and at the end of the academic year) on each outcome. The adjusted model adjusts for two factors: i) whether or not the participants enrolled in graduate school the same year they graduated from an undergraduate institution; ii) whether or not they attended a Historically Black College or University, a Hispanic Serving Institution, or other Minority Serving Institution (compared to a predominantly white institution) for their undergraduate education. Neither of these control variables were significantly associated with any of the outcomes at p <.05. The *F_time_* is the F value for the overall effect of the Hidden Curriculum event over time (pre-Hidden Curriculum, post-Hidden Curriculum, and at the end of the academic year); the p value (time) column shows the significance of that effect. The *M_diff_* columns show the mean difference in scores from pre-Hidden Curriculum to post-Hidden Curriculum, from pre-Hidden Curriculum to the end of the year, and from post-Hidden Curriculum to the end of the year; the p value (*M_diff_*) column shows the significance of those differences. Effects significant at p < .05 are bolded.
